# Universal Framework for Multiconfigurational DFT

**DOI:** 10.1021/acs.jctc.4c01687

**Published:** 2025-03-07

**Authors:** Mickael G. Delcey

**Affiliations:** Division of Computational Chemistry, Department of Chemistry, Lund University, SE-221 00 Lund, Sweden

## Abstract

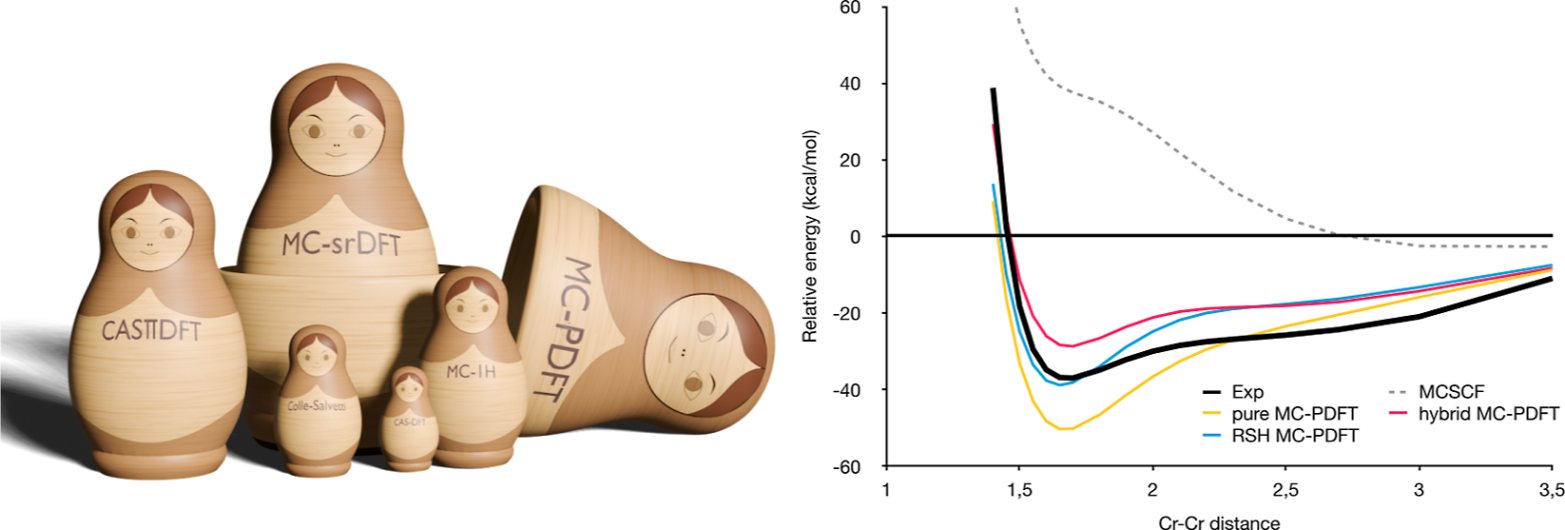

Strong correlation remains a significant challenge for DFT with no satisfying solutions found yet within the standard Kohn–Sham framework. Instead, for decades, a number of different approaches have been suggested to combine the accuracy of multiconfigurational methods with the efficiency of DFT. In this article, we demonstrate that many of these methods are or would be significantly improved by being reformulated as variants of multiconfigurational pair-density functional theory (MC-PDFT). This work presents the first implementation of these methods within the recently proposed variational formulation of MC-PDFT. It also provides for the first time a systematic comparison of their accuracy across representative examples of strongly correlated systems. By analyzing their accuracy and formal properties, we provide design guidelines to inform the development of future functionals.

## 1 Introduction

Most widely used quantum chemistry methods rely on the assumption that the electronic structure of a system can be qualitatively described by a single electronic configuration. This assumption is explicit in post-Hartree–Fock methods such as Møller–Plesset^1^ or coupled cluster which rely on a Hartree–Fock wave function as the reference state. In Kohn–Sham density functional theory (KS-DFT),^2^ the energy is formally exact even with a single configuration. However, in practice, approximate spin-density functionals cause KS-DFT to behave like a single-configurational method.

Although this single-configuration assumption works well for a surprisingly large part of chemistry, it breaks down in many important cases. In particular, in transition metal complexes^3^ or in photochemistry, especially around crossing points such as conical intersections,^4−6^ the wave function often consists of a number of significant electron configurations, rendering single-configurational methods unreliable. In such cases, the proper methods to use are multiconfigurational methods. There has recently been an increased focus on these methods, and several perspectives have highlighted their growing applicability to real-scale problems.^7,8^

Multiconfigurational methods generalize standard single-configurational approaches. One of the simplest and least expensive of these is the Multiconfigurational Self-Consistent Field (MCSCF),^9,10^ which serves as the multiconfigurational counterpart to Hartree–Fock. To add dynamical correlation, we can then use multireference perturbation theory such as the complete active space perturbation theory (CASPT2)^11^ and the N-electron valence perturbation theory (NEVPT2),^12^ which are generalizations of Møller–Plesset (MP2). Other approaches include multireference extensions of the algebraic diagrammatic construction,^13^ configuration interaction,^14^ and coupled cluster.^15^ However, the cost of even the cheaper multireference perturbation theory is often prohibitive for large-scale applications. This comes from its N^5^ formal scaling with system size but also the need for high-order density matrices in the active space. To make things worse, for geometry optimization or (nonadiabatic) ab initio molecular dynamics, analytical gradients have only recently been derived^16−18^ and are still not broadly available.

As mentioned earlier, KS-DFT behaves essentially like a single-configurational method, and no satisfying approaches have been found within this framework to solve the strong correlation issue.^19^ This problem has been dubbed by some “the last frontier in DFT“.^20,21^ Motivated by the success of KS-DFT for single-configurational systems, significant efforts have been made to extend DFT to multiconfigurational wavefunctions. This has resulted in a real zoo of methods each with their own strengths and weaknesses which we will collectively refer to as multiconfigurational DFT (MC-DFT).^22−34^ This plethora of methods has dispersed the efforts of the community and prevented the emergence of a standard.

In this article, we address this issue by reviewing several of these approaches. By analyzing their formal properties, we demonstrate that many of these methods benefit from being reformulated as special cases of the multiconfigurational pair-density functional theory (MC-PDFT),^32−34^ simply becoming different MC-PDFT functionals. Combined with our recently developed variational formulation of MC-PDFT,^34^ we show that several of these functionals exhibit all desirable formal properties expected of a multiconfigurational DFT. We also present, for the first time, a benchmark comparison of these functionals on representative strong correlation cases. From this analysis, we draw a useful conclusion to guide the design of improved functionals.

## 2 Theory

### 2.1 Formal Properties

To compare existing approaches, it is useful to first establish a set of formal properties that an ideal multiconfigurational DFT method should possess. One key property is formal exactness, akin to the role of the Hohenberg–Kohn theorem^35^ in standard DFT. While not directly a guarantee of accuracy, this assures that the search for improved functionals is meaningful. In multiconfigurational DFT, the notion of formal exactness is complicated by the presence of an active space. Should formal exactness require a full CI expansion or should it hold for any chosen active space? A stricter property is convergence to the full CI result as we increase the active space regardless of the functional. This is a common property in a multireference method, such as CASPT2 or NEVPT2, MRCI, etc. However, for MC-DFT, it may be undesirable, as the method would inherit the poor basis set convergence of full CI rather than the faster convergence of DFT.^36^ Another desirable property is that the method should be reduced to standard Kohn–Sham DFT for a single determinant. It is also a common feature of multiconfigurational methods; for example, CASPT2 and NEVPT2 reduce to MP2 when the reference space contains a single configuration. While not strictly necessary, it ensures that we inherit some of the most positive aspects of standard DFT. It is also important for the method to be able to describe correctly both ground and excited states, especially as photochemistry and spectroscopy are some of the most important application areas of multiconfigurational methods. Finally, it is highly desirable for the method to be variational, particularly for property calculations. It is a reasonable expectation, as both DFT and MCSCF are variational.

While these properties are valuable, they are not decisive. For instance, several techniques exist to simulate excited states from a given electronic structure method, such as response theory, state averaging, or Δ-SCF, with the quality of the results often being as dependent on the technique as on the underlying method itself. Similarly, many methods were not initially variational, but this can often be addressed, making it a secondary concern. Therefore, we focus on properties that are intrinsically tied to the method formulation, namely

1. it should correctly dissociate bonds
2. it should work equally well for any spin multiplicities
3. it should be free from double-counting

The first property is a basic requirement for correctly describing strong correlation, assuming an appropriate active space. In practice, this property can be challenging to evaluate. A simpler criterion is that during a homolytic dissociation, the energy should converge to the sum of the fragment energies computed separately, without introducing any symmetry breaking. This is of course conditional on a consistent active space selection, which can have a degree of arbitrariness when comparing the subsystems to the full system. The second property, while seemingly obvious, is often overlooked. Many MC-DFT approaches are formulated around singlet closed-shell densities and are not easily generalized to other spin multiplicities. The final property addresses a frequent issue in MC-DFT methods that combine the MCSCF and DFT contributions. In such a method, the MCSCF energy captures an increasing fraction of correlation as the active space grows, potentially double counting the correlation already included in the DFT component.

With these properties in mind, we can now give a short review of the field.

### 2.2 Historical Developments

The very first proposed MC-DFT was by Lie and Clementi,^22^ with a simple scheme consisting of the sum of the MCSCF energy with a DFT correlation functional
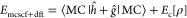
1with |MC⟩ a multiconfigurational wave function and *ĥ* and *ĝ* the one- and two-electron part of the electronic Hamiltonian operator, respectively.

The core motivation for combining MCSCF and DFT in such a way is to leverage their complementary strengths: MCSCF captures exchange and strong correlation, while DFT efficiently handles dynamical correlation. A major issue, however, is the potential for double counting, as the MCSCF wave function often captures part of the dynamical correlation, especially for larger active spaces. To mitigate this, the original work by Lie and Clementi restricted their calculations to a maximum of three configurations. Their approach relied on an older DFT functional based solely on the total density. While this ensured correct bond dissociation—the primary goal of the study—it lacked the capability to accurately describe systems with varying spin multiplicities.

This seminal paper inspired significant efforts in the field to address the issue of double counting. Many subsequent approaches introduced an additional parameter in the functional to quantify the correlation already captured by the MCSCF wave function. One prominent family of methods, often referred to as CASDFT, estimates the correlation by using the inactive density and/or the density of fully occupied active orbitals.^23−25,37^ This can be used to design a functional scaling down the part of the correlation treated by DFT as the size of the active space increases.

Another family of methods in this category makes use of the on-top pair density as a measure of the existing correlation. Indeed, the on-top pair density corresponds to the probability of two electrons being in the same point in space and is thus directly linked to correlation. This idea can be argued to date back from the pioneering work of Colle and Salvetti who derived an on-top pair-density correlation functional, first for closed-shell cases^38^ but then extended to open shells and multiconfigurational wave functions.^26,39^ In these later articles, they introduced a term in their on-top pair-density functional meant to attenuate the correlation as the quality of the wave function improved. Similar ideas have been suggested, for example, in the work of Gusarov et al.,^27^ although they did not provide any such functional, and later, the same motivating concept was put in practice in the so-called CASΠDFT method.^28,40^

A widely studied multiconfigurational DFT approach involves range separation.^29−31^ In this scheme, double counting is strictly avoided thanks to the range separation of the two-electron repulsion term
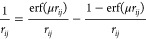
2
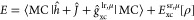
3where we defined the exchange–correlation two-electron operator , with *Ĵ* the Coulomb operator.

The rationale for range separation lies in the observation that dynamical correlation is predominantly short-ranged, well-handled by DFT, while strong correlation often arises at a long range, as in bond dissociations. In terms of formal properties, this method is formally justified and variational, matches standard range-separated hybrid DFT for a single configuration, and is free from double counting. However, the DFT contribution, though smaller, still brings its usual challenges. In the original implementation, the functional depended only on the total density, which kept it from being able to accurately describe open shells. In a more recent work,^41^ a spin-density formulation was proposed instead, but this now hinders a proper description of dissociations.

Finally, there has been much less work on hybrid functionals with a smaller portion of MCSCF energy, probably due to the difficulty in describing the exchange energy at the DFT level without compromising the description of strong correlation before the advent of PDFT. The main milestone in this category is the work by Sharkas et al.^42^ They formulate their approach as a form of double hybrid where, to avoid double counting, both the DFT exchange and the correlation are scaled down. When including a portion λ of MCSCF energy, the DFT exchange is scaled by (1 – λ) as in standard hybrids, but by contrast to many common double hybrids, they argue that the DFT correlation should be scaled by a factor of (1 – λ^2^) and use a scaled density. The argument was based on a perturbation expansion of the energy where the correlation is at least the second order. For simplification, they also showed that the density scaling does not significantly affect the result and proposed a simpler multiconfigurational one-parameter hybrid
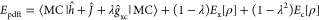
4

This approach is variational and free of double counting. However, due to using a density-only functional, it could not describe open shells correctly.

It should be noted that while the scaling prevents double counting, it threatens to underestimate the correlation. This formulation assumes that the MCSCF includes all correlation, i.e., full CI. It is easy to see that for a single determinant, the scaling of the correlation functional will not be compensated by the (absent) MCSCF correlation. Similarly, setting λ to 1 in this formula would simply remove all DFT correlation and give back the MCSCF energy, which we know typically lacks most of the dynamical correlation. However, due to the λ^2^ factor, the resulting error is reasonably small for the typical values of λ. Assuming a 20% MCSCF exchange, in line with the usual DFT hybrids, the reduction of DFT correlation would be only 4%.

### 2.3 MC-PDFT

MC-PDFT is unique among multiconfigurational methods due to its foundation within a purely DFT framework. To understand its concept, it is important to first explore why traditional DFT struggles to capture a strong correlation.

Formally, any reasonably local functional of the total density fulfills property 1. This may seem surprising, as most practical implementations of DFT codes instead lead to a significant discrepancy. This discrepancy arises because most modern DFT implementations rely on spin-density functionals instead of only functionals of the total density.

While the Hohenberg–Kohn theorem asserts that the total density should be sufficient, spin-density functionals are commonly used in practice. These provide an intuitive and more accurate description of the exchange energy, especially for open-shell systems. However, such functionals can provide consistent homolytic dissociation energies only by breaking spin symmetry. This comes with numerous issues and is particularly problematic when using a multiconfigurational wave function since the spin symmetry is usually respected there. This means that a naive multiconfigurational DFT with modern spin-density functionals does not give consistent homolytic dissociation.

A compelling solution to this issue is to replace the spin polarization with the on-top pair density.^43,44^ For a single determinant, there is a one-to-one relation between spin densities and the on-top pair density. This allows us to “translate” spin-density functionals to use the on-top pair density of a multiconfigurational wave function instead while providing the same result for a single determinant. However, the on-top pair density is a well-defined quantity that remains consistent in a dissociation or when describing fragments separately.

This concept was then fully implemented under the name MC-PDFT.^32^ In this approach, the energy is computed by a Kohn–Sham-like formula
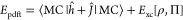
5with *E*_xc_ a functional of the total density ρ and on-top pair density Π, which are both computed from the multiconfigurational wave function.

MC-PDFT can be formally justified by generalizing the Levy constrained search formulation^45^ which provides a rigorous definition of the exact universal pair-density functional
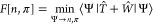
6This makes PDFT formally exact, although achieving the exact result would still require a full CI expansion in a complete basis set. This theory can describe all spin multiplicities and unlike standard DFT, it preserves the degeneracy of all *m*_s_ components of a given spin (prop. 2). In addition, because the energy is not the sum of a DFT and a MCSCF contribution, there is formally no double counting of the electron correlation (prop. 3).

Initial implementations of MC-PDFT relied on wave functions optimized with MCSCF. This approach introduced several issues. In particular, it meant that the wave function was optimized without the influence of correlation. In extreme cases, the wave function could even converge to the wrong state. Not being variational made property calculations more challenging. It also meant the result does not match the corresponding Kohn–Sham for a single determinant. We have previously shown that MC-PDFT can be implemented variationally to obtain the multiconfigurational wave function that minimizes the MC-PDFT energy.^34^ This new formulation MC-PDFT can really be seen as an extension of Kohn–Sham DFT for multiconfigurational wave functions. It retains the correct single-configuration limit while addressing bond dissociation without artificial symmetry breaking (prop. 1). Our variational MC-PDFT thus satisfies all formal properties we proposed earlier.

We note, however, that this defines only pure functionals, i.e., without MCSCF exchange and correlation. As MC-PDFT extends standard DFT, it is reasonable to assume that the currently available pure functionals may not be accurate enough in many applications, and our initial benchmarks confirmed this. The development of hybrid functionals, inspired by earlier multiconfigurational schemes, offers a promising path forward.

### 2.4 Unifying the Frameworks

All of the approaches discussed in Section 2.2 incorporate at least a fraction of MCSCF energy added to a DFT term. While this MCSCF component correctly describes both dissociation (prop. 1) and spin (prop. 2), the DFT term inevitably fails at least one of these properties depending on whether it uses the total density only or the spin densities. None of these frameworks, therefore, fully satisfies all desired properties. However, replacing the DFT term with a pair-density functional resolves these limitations without altering the essence of the methods, making this substitution a strict improvement.

This has already been done by some groups for a number of these methods. First, despite its name, CASΠDFT is already a hybrid form of MC-PDFT with 100% MCSCF. Similarly, several CASDFT implementations also made use of on-top pair-density functionals,^24,25^ though they cannot be considered special cases of MC-PDFT due to their additional functional arguments and are thus excluded here. Still, these formulations are straightforward to implement within the MC-PDFT framework. The multiconfigurational one-parameter hybrid has also been extended to MC-PDFT by two different groups^46,47^ and is now the main formulation for global hybrid functionals for MC-PDFT. It is worth noting that these formulations are not variational. This is especially problematic as the (1 – λ^2^) scaling argument is only valid if the wave function is optimized with a λ fraction of MCSCF correlation in the Hamiltonian. When using the full MCSCF during the wave function optimization, as done in these implementations, the scaling should be (1 – λ), but using this factor leads to a more severe underestimation of correlation. This issue is avoided in our variational implementation. More recently, our colleagues adapted a pair-density functional into their range-separated MC-srDFT, albeit initially limited to a translated LDA functional.^48^ This formulation retains all of the desirable properties of MC-PDFT and represents a range-separated hybrid functional variant of the method.

We note that this discussion excludes some methods. For example, density-matrix functional theory^49^ has shown promise in treating strong correlation but falls outside the scope of this article due to its lack of explicit multiconfiguration wave function. Similarly, the recently suggested multiconfiguration density-coherence functional theory^50^ is excluded as it has the same issue as DFT with functionals of only the total density. While it can dissociate properly, the density coherence cannot easily distinguish a triplet from the corresponding open-shell singlet since they have in many cases (near-)identical occupation numbers.

With these exceptions, it follows that nearly all existing multiconfigurational DFT can be unified under a single framework as different MC-PDFT functionals. Since this unification enhances their fundamental properties, it should be the preferred approach. These functionals can be straightforwardly implemented within a unified codebase, offering a cohesive and versatile platform for addressing strong electron correlation.

### 2.5 Final Functionals

We adapted many of these schemes as new functionals in our variational MC-PDFT implementation in MultiPsi.^51^ This is the first time all of the schemes are made available in the same program, and for many of them, it is the first time they are implemented in a variational framework.

For pure MC-PDFT functionals, we used the already described complex translation ctLDA, ctPBE, and ctBLYP from ref (52). For the MCSCF + DFT hybrids, we implemented a scheme similar to that of Lie and Clementi by simply adding the ctLYP functional to the full MCSCF energy, which will inevitably result in double counting. We also used the HPG20 CASΠDFT functional,^40^ which is based on LYP but with an additional factor designed to remove the double counting. For the global hybrids, we use the translated version of B3LYP and PBE0, using the MC-1H scaled correlation, which we call ctB3LYP and ctPBE0.

Finally, for range-separated functionals, we used the corresponding functionals implemented with the erf attenuation function. We implemented srLDA, corresponding to the functional from Paziani, Moroni, Gori-Giorgi, and Bachelet (PMGB06),^53^ itself using the exchange functional from Toulouse et al.,^54^ as well as the short-range PBE, using the definition from ref (55). For the short-range BLYP, we used the exchange from Iikura, Tsuneda, Yanai, and Hirao^56^ and the correlation from Ia, Fang, and Sun.^57^ The range-separation parameter was set to 0.4 for srLDA and 0.33 for srPBE and srBLYP. These short-range functionals were translated, including in the complex regime, in order to become pair-density functionals, as described in ref (52). However, for the short-range exchange, no analytical expression could be found using real arithmetics due to the presence of the erf function, and instead, we used
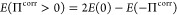
7to translate cases where the “correlated” on-top pair density  was positive (and thus, the translated spin polarization would be imaginary).

While we implemented all of these functionals, our benchmarking below will focus on the ones from the BLYP family to focus more on the effect of the scheme than the individual functional performance.

## 3 Computational Details

In order to assess the accuracy of the different functionals, we compared them on three main tests relating to the three key properties.

The first test focuses on spin and is the small singlet–triplet benchmark set we already used in our previous paper on functional translation.^52^ This set contains both Hund-obeying molecules (meaning the triplet is lower than the corresponding singlet) as well as anti-Hund (open-shell singlet lower than the triplet). The reference values are the doubly electron-attached coupled-cluster data^58^ for the conjugated organic systems and experimental data for the smaller molecules.^59^ We use the same minimal and extended active space (so-called π active space since it contains π orbitals) as we did then, but calculations were run on a larger basis, namely, def2-tzvp.^60^

The second test assesses the double counting by simulating small systems with increasing active spaces from the minimal (single determinant) to full CI. Bond energies are particularly sensitive to the amount of correlation, and thus, we chose B_2_ bond dissociation as an example, using the def2-svp^60^ basis. The active space for B_2_ consists of a minimal cas(2,2) which is a single determinant since the molecule is a triplet, a standard cas(6,8) including 2s and 2p orbitals, and the full CI keeping the 1s orbitals frozen.

Finally, the last test focuses on a full dissociation profile, and for this, we used the challenging case of chromium dimer, for which the experimental results were recently reinterpreted with the help of sophisticated simulations.^61^ We use the standard cas(12,12) active space and the def2-tzvp basis.

All calculations were performed with MultiPsi.^51,62^ The default grid size for the GGA functionals (grid level 4) was used throughout.

## 4 Results and Discussion

### 4.1 Spin

The correct description of spin energetics is one of our key properties. To assess this, we use a small benchmark set composed of small molecules and some antiaromatic organic molecules. Most of the gaps considered are between triplet and open-shell singlet states, which can be classified as either Hund or anti-Hund, depending on whether the triplet state is lower in energy than the singlet. The description of anti-Hund cases is particularly challenging for DFT,^63^ and our previous studies have shown disappointing results for pure PDFT.^34^

**Figure 1:**
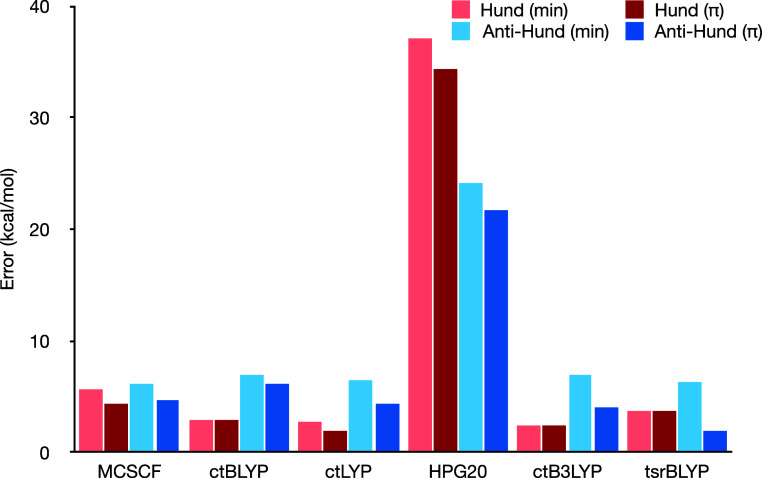
Errors on the singlet–triplet gaps for various PDFT functionals for the minimal (2,2) and π active space, separating the Hund-obeying and non-Hund-obeying molecules. The reference values are from high-level theory^58^ or experiments.^59^

Figure 1 presents the errors in the PDFT-predicted gaps, separated into Hund and anti-Hund cases, while Figure 3 shows the combined errors (with larger errors truncated for clarity).

**Figure 2:**
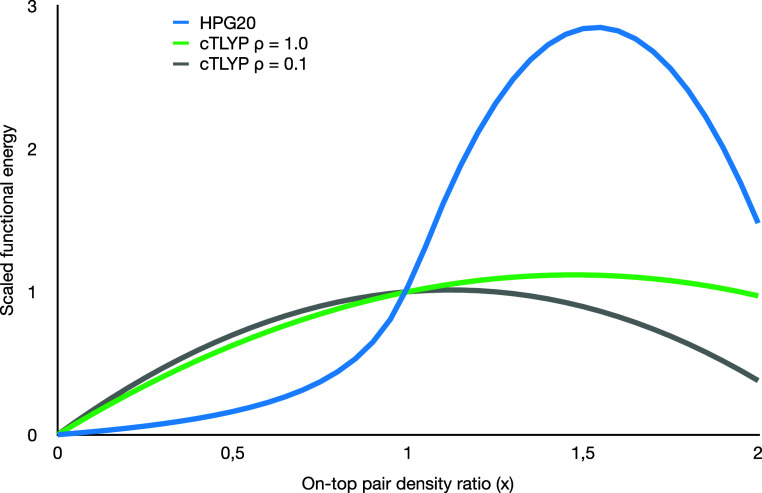
Comparison of the pair-density dependence of CASΠDFT (functional HPG20) and ctLYP for two different densities. We use the on-top pair density *x* defined in ref (28).

**Figure 3:**
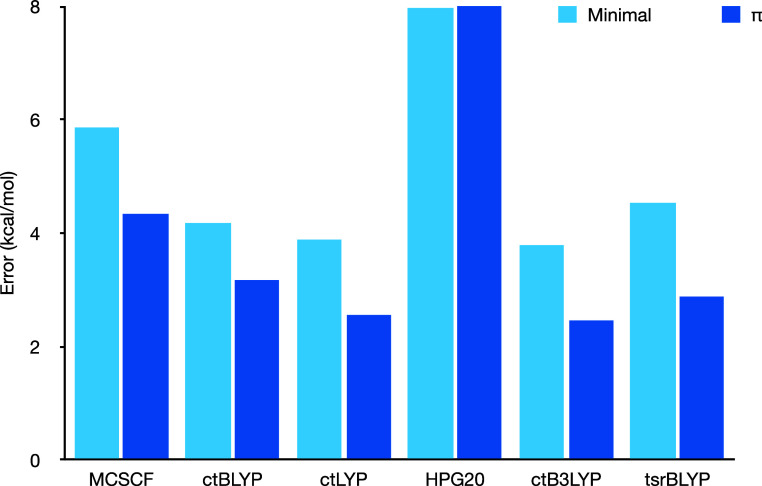
Errors on the singlet–triplet gaps for various PDFT functionals for the minimal (2,2) and π active space, combining all molecules. Note that the HPG20 error is truncated for more visibility.

The most striking feature in Figure 1 is the significant error in the HPG20 functional. To understand this, we can look into how the functional was designed. The pair-density functional of HPG20 is expressed as a product of a standard correlation functional (LYP in this case) computed without spin polarization and a pair-density-dependent factor. This factor scales down the functional contribution as more correlation is included in the wave function. However, the pair-density term also models the spin dependence as well as the enhancement of correlation for low-spin open-shell cases, similarly to what we discussed in our previous work on PDFT functional translation.^52^

It is interesting to compare the pair-density dependence of our translated LYP functional with the CASΠDFT functional HPG20 (ref (40)), see Figure 2.

The broad qualitative trends are similar, including the absence of a DFT correlation at *x* = 0 and the existence of a maximum in the *x* > 1 region. *x* = 1 corresponds to a closed-shell single determinant wave function, where the pair-density scaling is by construction 1. However, both attenuation (*x* < 1) and enhancement (*x* > 1) are much more pronounced in HPG20. A stronger attenuation may at first seem reasonable, as CASΠDFT aims to remove the correlation already accounted for in the active space. However, the behavior of ctLYP for *x* ≤ 1 is based on the spin-polarized behavior, and thus, the deviation from it in HPG20 represents a potential error for high-spin cases. Since our test cases involve computing the energy difference between the triplet (*x* < 1) and open-shell singlet (*x* > 1), the exaggerated features of the HPG20 functional strongly impact the results. The singlets end up significantly stabilized, and the triplets substantially destabilized, leading to consistently large errors in the predicted singlet–triplet gaps.

Beyond HPG20, we see that generally, most PDFT results are better for the Hund molecules than anti-Hund ones, particularly when using a minimal active space. Actually, none of the functionals predicted the anti-Hund behavior for this active space. However, when using a larger active space, all hybrid functionals (i.e., all but ctBLYP) correctly predicted the anti-Hund behavior (with one exception for ctB3LYP in C_4_H_3_–NH_2_ which ends up with the wrong sign by about 0.6 kcal/mol). We note that MCSCF also correctly predicts the anti-Hund behavior in these cases. Still, this is a notable achievement since, as we wrote earlier, this is a tricky property to predict for DFT.

In general, all functionals benefit from the larger active space, with ctBLYP showing the smallest difference. Looking at the entire average, we see that ctB3LYP performs best, although tsrBLYP and ctLYP are not far behind.

### 4.2 Double Counting

**Figure 4:**
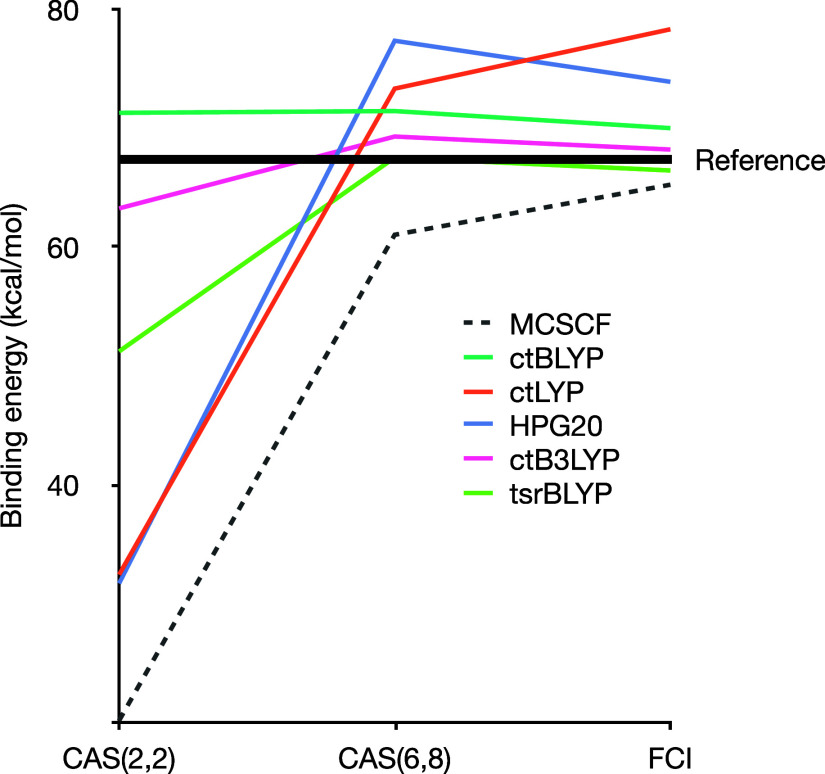
B_2_ binding energy for increasing active space size with various PDFT functionals.

To assess the existence and impact of double counting on the PDFT results, we computed the binding energy of B_2_ with various active space sizes. For reference, we indicate the near-full CI result extrapolated to the complete basis set of 67.7 kcal/mol.^64^ The results are shown in Figure 4.

This figure provides a wealth of information about the qualitative behavior of the various functionals. First, as expected, MCSCF converges toward the reference, though it falls slightly short due to the relatively small basis set. The span of the MCSCF result is also quite wide, with more than 40 kcal/mol error for the minimal active space (which corresponds to restricted open-shell Hartree–Fock). Since B_2_ is known to exhibit strong correlation effects, the poor performance of single-determinant methods on such a small molecule is unsurprising.

Simply adding a correlation functional (ctLYP) essentially introduces a constant shift toward higher binding energy. While this improves the result for small active spaces, it quickly leads to overbinding, already becoming excessive in the standard cas(6,8) active space. This is a clear example of the double-counting error. Interestingly, the HPG20 functional does not fare much better. While some correlation attenuation is evident in the full CI result, it is clearly insufficient. However, this is to a large extent because the molecules are in the triplet state. As shown in Figure 2, the attenuation is the strongest around *x* = 1, that is, from a closed-shell singlet. Starting from a triplet (i.e., *x* already lower than 1), the attenuation is much less steep. This highlights again the fundamental challenge with using a pair-density scaling functional to remove double-counting: the pair density already describes spin polarization and the two behaviors are quite different.

The pure MC-PDFT functional behaves very differently from the MCSCF, showing little dependence on the active space size. This trend has been observed in previous studies for nonvariational MC-PDFT^65^ and is even more pronounced in the variational framework.^34^ However, these functionals tend to overbind, a common issue with pure functionals even in standard DFT. Introducing a fraction of MCSCF energy, either through a global or range-separated hybrid, solves the overbinding at the cost of reintroducing a higher active-space dependence. Nevertheless, already with the standard cas(6,8), the results are nearly converged and in good agreement with the reference. As expected, no double-counting is visible here, and any potential undercounting in ctB3LYP is too minor to be noticeable. Overall, the two hybrids ctB3LYP and tsrBLYP perform best, provided that an appropriate active space is chosen.

### 4.3 Dissociation

**Figure 5:**
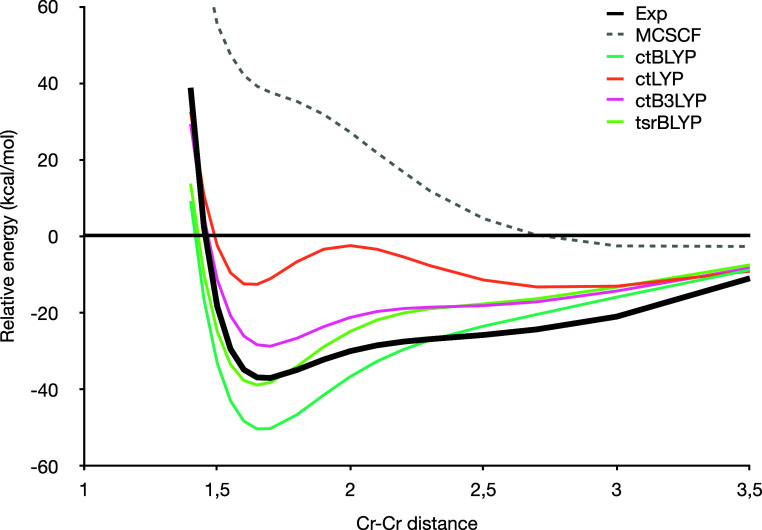
Cr_2_ dissociation profile with various PDFT functionals compared to the revised experimental profile of ref (61). The energies are relative to the dissociation limit computed as twice the energy of a chromium atom.

We end with one of the most famous and challenging dissociation profiles, namely, the chromium dimer. This system is notoriously difficult due to the presence of strong correlation along the entire dissociation curve (even near equilibrium). But even dynamical correlation is very significant, and as a result, MCSCF alone barely predicts binding. The results for the various functionals are shown in Figure 5. The HPG20 result is absent because the wave function failed to converge. This issue arises from a discontinuity in the second derivative at *x* = 1 that caused difficulties for our second-order CI algorithm.

First, as expected, all functionals correctly approach the correct dissociation limit, consistent with property 1. However, the spread of results is fairly wide. As described earlier, the pure PDFT functional (ctBLYP) overbinds. The range between ctBLYP and MCSCF results suggests that hybrid functionals would do well, and indeed, both ctB3LYP and tsrBLYP yield improved binding energies. tsrBLYP is the closest to the experiment, but the shape of the potential is slightly too deep. In contrast, ctB3LYP runs almost parallel to the experimental curve but slightly underbinds. It is useful to note that the ctB3LYP result is significantly better than the one reported for the nonvariational scheme and on par with the ad-hoc tB4LYP suggested in a recent publication.^66^ This confirms the significant advantage of the variational formulation, especially when correlation is significant. Interestingly, despite concerns about double counting, ctLYP severely underbinds. This is also consistent with the standard Kohn–Sham experience. Despite ongoing research, it remains difficult to devise an accurate functional with 100% Hartree–Fock exchange. This challenge arises in part because the DFT exchange captures some effects that mimic correlation (in particular, strong correlation). Even with this cas(12,12) active space, the lack of DFT exchange penalizes the ctLYP functional, especially around the correct binding distance.

## 5 Conclusions

Throughout our results, it is clear that PDFT functionals behave largely as expected based on our experience with Kohn–Sham DFT and the formal properties discussed earlier. Pure MC-PDFT has a number of attractive properties, especially its lower computational cost and minimal active space dependence. Yet, while its performance is reasonable, it falls short of the desired chemical accuracy.

On the opposite side of the spectrum, hybrid functionals with 100% MCSCF energy, despite being historically the most common forms of multiconfigurational DFT, are clearly inadequate. They suffer from double-counting, which is difficult to correct using a pair-density-dependent scaling term without compromising spin behavior. HPG20 was parameterized empirically for the nonvariational approach and would likely require reparameterization for variational use. However, this would not resolve the fundamental conflict between spin dependence and the double-counting correction. Additionally, as in Kohn–Sham DFT, achieving quantitative accuracy without DFT exchange remains a challenge.

The most promising approaches are hybrid functionals with a moderate MCSCF contribution (around 20% as is standard in KS-DFT) or range-separated hybrids. Like pure PDFT, these approaches fulfill all of the desirable formal properties. While global hybrids introduce a potential risk of undercounting, the λ^2^ dependence makes this manageable. Our benchmark results demonstrate their great potential for quantitative accuracy at the price of a slightly increased cost and active space dependence compared to the pure functionals.

More broadly, this study highlights MC-PDFT as the most promising framework for most multiconfigurational DFT. Its flexibility allows it to unify a wide range of approaches, bringing to them appealing formal properties and increased predictive accuracy. We hope this work will encourage the community to adopt MC-PDFT as a standard framework and shift focus toward the development of more accurate functionals, for which our findings provide useful guidelines.

## References

[ref1] Møller C.; Plesset M. S. Note on an Approximation Treatment for Many-Electron Systems. Phys. Rev. 1934, 46, 618–622. 10.1103/PhysRev.46.618.

[ref2] Kohn W.; Sham L. J. Self-Consistent Equations Including Exchange and Correlation Effects. Phys. Rev. 1965, 140, A1133–A1138. 10.1103/PhysRev.140.A1133.

[ref3] Khedkar A.; Roemelt M. Modern multireference methods and their application in transition metal chemistry. Phys. Chem. Chem. Phys. 2021, 23, 17097–17112. 10.1039/D1CP02640B. 34355719

[ref4] Levine B. G.; Ko C.; Quenneville J.; MartÍnez T. J. Conical intersections and double excitations in time-dependent density functional theory. Mol. Phys. 2006, 104, 1039–1051. 10.1080/00268970500417762.

[ref5] Winslow M.; Cross W. B.; Robinson D. Comparison of Spin-Flip TDDFT-Based Conical Intersection Approaches with XMS-CASPT2. J. Chem. Theory Comput. 2020, 16, 3253–3263. 10.1021/acs.jctc.9b00917. 32302484 PMC8279405

[ref6] Barneschi L.; Kaliakin D.; Huix-Rotllant M.; Ferré N.; Filatov(Gulak) M.; Olivucci M. Assessment of the Electron Correlation Treatment on the Quantum-Classical Dynamics of Retinal Protonated Schiff Base Models: XMS-CASPT2, RMS-CASPT2, and REKS Methods. J. Chem. Theory Comput. 2023, 19, 8189–8200. 10.1021/acs.jctc.3c00879. 37937990

[ref7] Vitillo J. G.; Cramer C. J.; Gagliardi L. Multireference Methods are Realistic and Useful Tools for Modeling Catalysis. Isr. J. Chem. 2022, 62, e202100136 10.1002/ijch.202100136.

[ref8] Jørgensen F. K.; Delcey M. G.; Hedegård E. D. Perspective: multi-configurational methods in bio-inorganic chemistry. Phys. Chem. Chem. Phys. 2024, 26, 17443–17455. 10.1039/D4CP01297F. 38868993

[ref9] Hinze J.; Roothaan C. C. J. Multi-Configuration Self-Consistent-Field Theory. Prog. Theor. Phys. Suppl. 1967, 40, 37–51. 10.1143/PTPS.40.37.

[ref10] Roos B. O.; Taylor P. R.; Sigbahn P. E. A complete active space SCF method (CASSCF) using a density matrix formulated super-CI approach. Chem. Phys. 1980, 48, 157–173. 10.1016/0301-0104(80)80045-0.

[ref11] Andersson K.; Malmqvist P. Å.; Roos B. O.; Sadlej A. J.; Wolinski K. Second-order perturbation theory with a CASSCF reference function. J. Phys. Chem. 1990, 94, 5483–5488. 10.1021/j100377a012.

[ref12] Angeli C.; Pastore M.; Cimiraglia R. New perspectives in multireference perturbation theory: the n-electron valence state approach. Theor. Chem. Acc. 2007, 117, 743–754. 10.1007/s00214-006-0207-0.

[ref13] Mazin I. M.; Sokolov A. Y. Multireference Algebraic Diagrammatic Construction Theory for Excited States: Extended Second-Order Implementation and Benchmark. J. Chem. Theory Comput. 2021, 17, 6152–6165. 10.1021/acs.jctc.1c00684. 34553937

[ref14] Werner H.; Knowles P. J. An efficient internally contracted multiconfiguration–reference configuration interaction method. J. Chem. Phys. 1988, 89, 5803–5814. 10.1063/1.455556.

[ref15] Paldus J.; Pittner J.; Čársky P. In Recent Progress in Coupled Cluster Methods: Theory and Applications; Cársky P., Paldus J., Pittner J., Eds.; Springer Netherlands: Dordrecht, 2010; pp 455–489.

[ref16] Győrffy W.; Shiozaki T.; Knizia G.; Werner H.-J. Analytical energy gradients for second-order multireference perturbation theory using density fitting. J. Chem. Phys. 2013, 138, 104104 10.1063/1.4793737. 23514462

[ref17] Park J. W.; Al-Saadon R.; Strand N. E.; Shiozaki T. Imaginary Shift in CASPT2 Nuclear Gradient and Derivative Coupling Theory. J. Chem. Theory Comput. 2019, 15, 4088–4098. 10.1021/acs.jctc.9b00368. 31244126

[ref18] Park J. W.; Shiozaki T. Analytical Derivative Coupling for Multistate CASPT2 Theory. J. Chem. Theory Comput. 2017, 13, 2561–2570. 10.1021/acs.jctc.7b00018. 28471661

[ref19] Verma P.; Truhlar D. G. Status and Challenges of Density Functional Theory. Trends Chem. 2020, 2, 302–318. 10.1016/j.trechm.2020.02.005.

[ref20] Becke A. D. Perspective: Fifty years of density-functional theory in chemical physics. J. Chem. Phys. 2014, 140, 18A301 10.1063/1.4869598. 24832308

[ref21] Mardirossian N.; Head-Gordon M. Thirty years of density functional theory in computational chemistry: an overview and extensive assessment of 200 density functionals. Mol. Phys. 2017, 115, 2315–2372. 10.1080/00268976.2017.1333644.

[ref22] Lie G. C.; Clementi E. Study of the electronic structure of molecules. XXI. Correlation energy corrections as a functional of the Hartree-Fock density and its application to the hydrides of the second row atoms. J. Chem. Phys. 1974, 60, 1275–1287. 10.1063/1.1681192.

[ref23] Savin A. A combined density functional and configuration interaction method. Int. J. Quantum Chem. 1988, 34, 59–69. 10.1002/qua.560340811.

[ref24] Miehlich B.; Stoll H.; Savin A. A correlation-energy density functional for multideterminantal wavefunctions. Mol. Phys. 1997, 91, 527–536. 10.1080/002689797171418.

[ref25] Gräfenstein J.; Cremer D. The combination of density functional theory with multi-configuration methods - CAS-DFT. Chem. Phys. Lett. 2000, 316, 569–577. 10.1016/S0009-2614(99)01326-3.

[ref26] Colle R.; Salvetti O. Generalization of the Colle–Salvetti correlation energy method to a many-determinant wave function. J. Chem. Phys. 1990, 93, 534–544. 10.1063/1.459553.

[ref27] Gusarov S.; Malmqvist P.-Å.; Lindh R.; Roos B. O. Correlation potentials for a multiconfigurational-based density functional theory with exact exchange. Theor. Chem. Acc. 2004, 112, 84–94. 10.1007/s00214-004-0568-1.

[ref28] Gritsenko O. V.; van Meer R.; Pernal K. Efficient evaluation of electron correlation along the bond-dissociation coordinate in the ground and excited ionic states with dynamic correlation suppression and enhancement functions of the on-top pair density. Phys. Rev. A 2018, 98, 062510 10.1103/PhysRevA.98.062510.

[ref29] Stoll H.; Savin A. In Density Functional Methods In Physics; Dreizler R. M., da Providência J., Eds.; Springer US: Boston, MA, 1985; pp 177–207.

[ref30] Leininger T.; Stoll H.; Werner H.-J.; Savin A. Combining long-range configuration interaction with short-range density functionals. Chem. Phys. Lett. 1997, 275, 151–160. 10.1016/S0009-2614(97)00758-6.

[ref31] Fromager E.; Toulouse J.; Jensen H. J. A. On the universality of the long-/short-range separation in multiconfigurational density-functional theory. J. Chem. Phys. 2007, 126, 074111 10.1063/1.2566459. 17328597

[ref32] Li Manni G.; Carlson R. K.; Luo S.; Ma D.; Olsen J.; Truhlar D. G.; Gagliardi L. Multiconfiguration Pair-Density Functional Theory. J. Chem. Theory Comput. 2014, 10, 3669–3680. 10.1021/ct500483t. 26588512

[ref33] Gagliardi L.; Truhlar D. G.; Li Manni G.; Carlson R. K.; Hoyer C. E.; Bao J. L. Multiconfiguration pair-density functional theory: A new way to treat strongly correlated systems. Acc. Chem. Res. 2017, 50, 66–73. 10.1021/acs.accounts.6b00471. 28001359

[ref34] Scott M.; Rodrigues G. L. S.; Li X.; Delcey M. G. Variational Pair-Density Functional Theory: Dealing with Strong Correlation at the Protein Scale. J. Chem. Theory Comput. 2024, 20, 2423–2432. 10.1021/acs.jctc.3c01240. 38217859 PMC10976634

[ref35] Hohenberg P.; Kohn W. Inhomogeneous Electron Gas. Phys. Rev. 1964, 136, B864–B871. 10.1103/PhysRev.136.B864.

[ref36] Kraus P. Basis Set Extrapolations for Density Functional Theory. J. Chem. Theory Comput. 2020, 16, 5712–5722. 10.1021/acs.jctc.0c00684. 32790303

[ref37] Nakata K.; Ukai T.; Yamanaka S.; Takada T.; Yamaguchi K. CASSCF version of density functional theory. Int. J. Quantum Chem. 2006, 106, 3325–3333. 10.1002/qua.21151.

[ref38] Colle R.; Salvetti O. Approximate calculation of the correlation energy for the closed shells. Theor. Chim. Acta 1975, 37, 329–334. 10.1007/BF01028401.

[ref39] Colle R.; Salvetti O. Approximate calculation of the correlation energy for the closed and open shells. Theor. Chim. Acta 1979, 53, 55–63. 10.1007/BF00547606.

[ref40] Hapka M.; Pernal K.; Gritsenko O. V. Local Enhancement of Dynamic Correlation in Excited States: Fresh Perspective on Ionicity and Development of Correlation Density Functional Approximation Based on the On-Top Pair Density. J. Phys. Chem. Lett. 2020, 11, 5883–5889. 10.1021/acs.jpclett.0c01616. 32589027 PMC7467739

[ref41] Hedegård E. D.; Toulouse J.; Jensen H. J. A. Multiconfigurational short-range density-functional theory for open-shell systems. J. Chem. Phys. 2018, 148, 214103 10.1063/1.5013306. 29884047

[ref42] Sharkas K.; Savin A.; Jensen H. J. A.; Toulouse J. A multiconfigurational hybrid density-functional theory. J. Chem. Phys. 2012, 137, 044104 10.1063/1.4733672. 22852594

[ref43] Moscardó F.; San-Fabián E. Density-functional formalism and the two-body problem. Phys. Rev. A:At., Mol., Opt. Phys. 1991, 44, 1549–1553. 10.1103/PhysRevA.44.1549. 9906119

[ref44] Becke A. D.; Savin A.; Stoll H. Extension of the local-spin-density exchange-correlation approximation to multiplet states. Theor. Chim. Acta 1995, 91, 147–156. 10.1007/s002140050094.

[ref45] Levy M. Universal variational functionals of electron densities, first-order density matrices, and natural spin-orbitals and solution of the v-representability problem. Proc. Natl. Acad. Sci. U.S.A. 1979, 76, 6062–6065. 10.1073/pnas.76.12.6062. 16592733 PMC411802

[ref46] Pandharkar R.; Hermes M. R.; Truhlar D. G.; Gagliardi L. A New Mixing of Nonlocal Exchange and Nonlocal Correlation with Multiconfiguration Pair-Density Functional Theory. J. Phys. Chem. Lett. 2020, 11, 10158–10163. 10.1021/acs.jpclett.0c02956. 33196208

[ref47] Mostafanejad M.; Liebenthal M. D.; DePrince A. E. I. Global Hybrid Multiconfiguration Pair-Density Functional Theory. J. Chem. Theory Comput. 2020, 16, 2274–2283. 10.1021/acs.jctc.9b01178. 32101416

[ref48] Jørgensen F. K.; Kjellgren E. R.; Jensen H. J. A.; Hedegård E. D. Multiconfigurational short-range on-top pair-density functional theory. arXiv 2024, arXiv:2409.05213 10.48550/arXiv.2409.05213. 39812248

[ref49] Pernal K.; Giesbertz K. J. Reduced density matrix functional theory (RDMFT) and linear response time-dependent RDMFT (TD-RDMFT). In Density-Functional Methods for Excited States; Spinger, 2016; pp 125–183. 10.1007/128_2015_624 25971917

[ref50] Zhang D.; Hermes M. R.; Gagliardi L.; Truhlar D. G. Multiconfiguration Density-Coherence Functional Theory. J. Chem. Theory Comput. 2021, 17, 2775–2782. 10.1021/acs.jctc.0c01346. 33818081

[ref51] Delcey M. G. MultiPsi: A python-driven MCSCF program for photochemistry and spectroscopy simulations on modern HPC environments. Wiley Interdiscip. Rev.: Comput. Mol. Sci. 2023, 13, e1675 10.1002/wcms.1675.

[ref52] Rodrigues G. L. S.; Scott M.; Delcey M. G. Multiconfigurational Pair-Density Functional Theory Is More Complex than You May Think. J. Phys. Chem. A 2023, 127, 9381–9388. 10.1021/acs.jpca.3c05663. 37889622 PMC10641845

[ref53] Paziani S.; Moroni S.; Gori-Giorgi P.; Bachelet G. B. Local-spin-density functional for multideterminant density functional theory. Phys. Rev. B: Condens. Matter Mater. Phys. 2006, 73, 155111 10.1103/PhysRevB.73.155111.

[ref54] Toulouse J.; Savin A.; Flad H.-J. Short-range exchange-correlation energy of a uniform electron gas with modified electron–electron interaction. Int. J. Quantum Chem. 2004, 100, 1047–1056. 10.1002/qua.20259.

[ref55] Goll E.; Werner H.-J.; Stoll H. A short-range gradient-corrected density functional in long-range coupled-cluster calculations for rare gas dimers. Phys. Chem. Chem. Phys. 2005, 7, 3917–3923. 10.1039/b509242f. 19810319

[ref56] Iikura H.; Tsuneda T.; Yanai T.; Hirao K. A long-range correction scheme for generalized-gradient-approximation exchange functionals. J. Chem. Phys. 2001, 115, 3540–3544. 10.1063/1.1383587.

[ref57] Ai W.; Fang W.-H.; Su N. Q. The Role of Range-Separated Correlation in Long-Range Corrected Hybrid Functionals. J. Phys. Chem. Lett. 2021, 12, 1207–1213. 10.1021/acs.jpclett.0c03621. 33482068

[ref58] Stoneburner S. J.; Shen J.; Ajala A. O.; Piecuch P.; Truhlar D. G.; Gagliardi L. Systematic design of active spaces for multi-reference calculations of singlet–triplet gaps of organic diradicals, with benchmarks against doubly electron-attached coupled-cluster data. J. Chem. Phys. 2017, 147, 164120 10.1063/1.4998256. 29096487

[ref59] Bao J. L.; Sand A.; Gagliardi L.; Truhlar D. G. Correlated-Participating-Orbitals Pair-Density Functional Method and Application to Multiplet Energy Splittings of Main-Group Divalent Radicals. J. Chem. Theory Comput. 2016, 12, 4274–4283. 10.1021/acs.jctc.6b00569. 27438755

[ref60] Weigend F.; Ahlrichs R. Balanced basis sets of split valence, triple zeta valence and quadruple zeta valence quality for H to Rn: Design and assessment of accuracy. Phys. Chem. Chem. Phys. 2005, 7, 3297–3305. 10.1039/b508541a. 16240044

[ref61] Larsson H. R.; Zhai H.; Umrigar C. J.; Chan G. K.-L. The Chromium Dimer: Closing a Chapter of Quantum Chemistry. J. Am. Chem. Soc. 2022, 144, 15932–15937. 10.1021/jacs.2c06357. 36001866 PMC9460780

[ref62] Rinkevicius Z.; Li X.; Vahtras O.; Ahmadzadeh K.; Brand M.; Ringholm M.; List N. H.; Scheurer M.; Scott M.; Dreuw A.; Norman P. VeloxChem: A Python-driven density-functional theory program for spectroscopy simulations in high-performance computing environments. Wiley Interdiscip. Rev.: Comput. Mol. Sci. 2020, 10, e1457 10.1002/wcms.1457.

[ref63] Kunze L.; Froitzheim T.; Hansen A.; Grimme S.; Mewes J.-M. Δ-DFT Predicts Inverted Singlet-Triplet Gaps with Chemical Accuracy at a Fraction of the Cost of Wave Function-Based Approaches. J. Phys. Chem. Lett. 2024, 15, 8065–8077. 10.1021/acs.jpclett.4c01649. 39083761

[ref64] Bytautas L.; Matsunaga N.; Scuseria G. E.; Ruedenberg K. Accurate Potential Energy Curve for B2. Ab Initio Elucidation of the Experimentally Elusive Ground State Rotation-Vibration Spectrum. J. Phys. Chem. A 2012, 116, 1717–1729. 10.1021/jp210473e. 22175225

[ref65] Sharma P.; Truhlar D. G.; Gagliardi L. Active Space Dependence in Multiconfiguration Pair-Density Functional Theory. J. Chem. Theory Comput. 2018, 14, 660–669. 10.1021/acs.jctc.7b01052. 29301088

[ref66] Feng R.; Zhang I. Y.; Xu X. A cross-entropy corrected hybrid multiconfiguration pair-density functional theory for complex molecular systems. Nat. Commun. 2025, 16, 235 10.1038/s41467-024-55524-z. 39747131 PMC11695591

